# Environmental Hazards of Giant Reed (*Arundo donax* L.) in the Macaronesia Region and Its Characterisation as a Potential Source for the Production of Natural Fibre Composites

**DOI:** 10.3390/polym13132101

**Published:** 2021-06-25

**Authors:** Luis Suárez, Jessica Castellano, Francisco Romero, María Dolores Marrero, Antonio Nizardo Benítez, Zaida Ortega

**Affiliations:** 1 Departamento de Ingeniería Mecánica, Universidad de Las Palmas de Gran Canaria, 35017 Las Palmas, Spain; jessica.castellano@ulpgc.es (J.C.); francisco.romero@ulpgc.es (F.R.); mariadolores.marrero@ulpgc.es (M.D.M.); 2 Departamento de Ingeniería de Procesos, Universidad de Las Palmas de Gran Canaria, 35017 Las Palmas, Spain; antonionizardo.benitez@ulpgc.es (A.N.B.); zaida.ortega@ulpgc.es (Z.O.)

**Keywords:** characterisation, natural fibres, *Arundo donax* L., giant reed, composites, NFC

## Abstract

This paper summarises the results obtained from the characterisation of giant reed (*Arundo donax* L.) plant and fibres. The research is part of a project developed in the Macaronesia region, of which the aim is to demonstrate the feasibility of using biomass from invasive plant species in the composites sector as a way of financing control campaigns and habitats conservation labours. An experimental procedure for the extraction of fibre bundles from this plant was developed, and the material obtained was characterised in terms of chemical composition, thermogravimetry and infrared spectra to evaluate its potential application in the production of polymeric composite materials as a strategy for the valorisation of residual biomass from this invasive species in Macaronesia. Thermoplastic matrix composites with fibre content up to 40 wt.% were produced and their mechanical properties under tensile, flexural and impact loading were determined. No references on the preparation of composite materials with polyolefin matrices and giant reed fibres have been found. Results obtained from mechanical tests show a good performance of the manufactured composites, with a significant increase in both flexural and tensile stiffness; the flexural modulus is almost tripled for PE-based composites and rises to 88% with respect to PP matrix. The ultimate flexural strength and the tensile and flexural yield strength are kept at acceptable values compared to neat polymer materials, although ultimate tensile strength and impact resistance are significantly affected when natural fibres are added.

## 1. Introduction

According to the catalogue of invasive species published by the International Union for Conservation of Nature (IUCN), *Arundo donax* L. is one of the 100 most dangerous plant species in the world due to its invasive nature and the alteration of the habitats it colonises [1,2].

This species, native from East Asia, is widely naturalised in the warm and tropical regions of the world. In the past, it was intentionally introduced to the Macaronesia archipelagos for its use in agriculture (fixing slopes, delimiting land), cattle raising and manufacturing of traditional tools. However, the loss of these traditional uses has favoured its progressive uncontrolled dissemination, producing the alteration of natural habitats and becoming an important risk factor in the spread of fires. This work assesses the potential use of giant reed specimens from the control and eradication campaigns of this invasive species, with the aim of supporting the maintenance of these environmental control strategies [3,4,5].

Several authors have reported the ecological risks associated with the uncontrolled growth of giant reed and its ability to promote the spread of wildfires and dominate ecosystems over native species. According to the monographic study on *Arundo* by Jimenez et al. [5], the difficulty in controlling the spread of this invasive species in the habitats it colonises lies in its morphology, its vegetative growth (via rhizome elongation and fragmentation) and its adaptability to a wide variety of environmental and climatic conditions, such as high salinity levels and long droughts. 

One of the most debated consequences of the giant reed invasion is fire, as its infestations increased fuel loads, as well as the frequency and intensity of wildfires. In addition to a high fuel load, *Arundo donax* stands have a tall and well-ventilated fuel structure that contains dry combustible all year. Even if only a limited percentage of the total biomass in the stand is dead and dry, it is sufficient to start and sustain wildfires [6]. Several studies have looked at post-fire forest regeneration and outlined strategies for giant reed removal. Coffman et al. [7] investigated the impact of wildfire on *Arundo donax* invasion in southern California by comparing its relative rate of reestablishment to native species following fire and discovered that *Arundo donax* grew 3–4 times faster than native woody riparian plants. In addition to increasing the risk of fire, the uncontrolled growth of this reed can disrupt natural land drainage and encroach on sensitive habitats [5].

As reported for several authors, giant reed has been used in wide range of applications, including raw material in biorefineries [8,9], eco‑friendly biosorbents [10,11,12], structural components and aggregates for bio-construction [13,14,15] and isolating panels [16,17], as well as in the composites industry as reinforcement phase [9,18,19,20]. García-Ortuño et al. have used chipped giant reed in a urea-formaldehyde matrix to produce compression moulded panels [21], with low thermal conductivity and mechanical properties suitable for furniture manufacturing. Fiore et al. [20] have processed the culm of giant reed and used it as fillers in PLA and epoxy resin matrices [18,22]; up to 15% for the epoxy composites (by infusion) and up to a 20% for PLA resin (by compression moulding). It was found that Young’s modulus improved due to the addition of the natural fibres, while the strength weakened for the epoxy composites. According to Scalici [19], plasma treatment seems to improve flexural properties of the epoxy composite (with a 5% fibre). When used as reinforcement in a polyurethane composite, NaOH treatment also increased the mechanical properties of the fibres, reaching up to 40% fibre loading.

Inv2Mac project (funded by Interreg MAC 2014–2020 program, grant number MAC2/4.6d/229) proposes to use the residual biomass from invasive species control to obtain natural fibres for their use in composites production. The primary goal of this project is to achieve a better environmental state of protection of natural habitats and biodiversity by observing the spread of various invasive plant species in Macaronesia, performing biological, chemical and mechanical characterisation and attempting to valorise them in obtaining composite materials. It is important to note that the aim of this research is not to produce economic profits, but to reduce waste generated by habitat maintenance actions and to invest the possible benefits on environmental restoration policies.

## 2. Materials and Methods

### 2.1. Plant Characterisation

**Figure 1:**
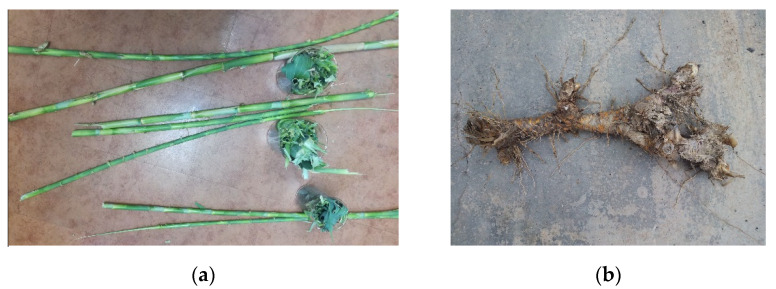
*Arundo donax* L.: (**a**) Stems, leaves and (**b**) roots.

Specimens of *Arundo donax* L. plants have been collected in an advanced stage of growth, with stems of an average diameter of 2 cm, in the area of the University Campus of Tafira (Gran Canaria, Canary Islands, Spain). The plants have been separated in three parts: Leaves, roots and stems (where most of fibres are found) for the characterisation tests (Figure 1). 

The lignin content was determined using the Klason method according to the ANSI/ASTM 1997a standard [23]. The analysis procedure consisted, basically, in a hydrolysis with H_2_SO_4_. The holocellulose content was determined following the technique described by Browning [24]: Acetic acid and sodium chlorite were used to degrade the lignin. 

Starting from the holocellulose sample, the determination of the total cellulose content was carried out following the ANSI/ASTM 1977b standard. The hemicellulose was calculated theoretically by the next expression:

Hemicellulose (%) = Holocellulose (%) − Cellulose (%)(1)

A total of 150 assays have been conducted, studying 3 different specimens, performing 5 replicas for each specimen in the different parts of the plant. Furthermore, thermogravimetric tests and surface chemical characterisation have been carried out on the different parts: stems, leaves and roots. 

The thermogravimetric tests were conducted in order to determine the thermal stability of the materials from the same parts in which was divided the plant, while the surface composition analysis was performed by Fourier transform infrared spectroscopy (FTIR). Thermogravimetrical analysis were performed in a Mettler Toledo TGA/DSC 1 device (Mettler Toledo, Columbus, OH, USA), from 25 to 1100 °C at a heating rate of 5 °C/min under nitrogen atmosphere. FTIR spectra were obtained in a Perkin Elmer spectrum Two device (Perkin Elmer, Waltham, MA, USA) equipped with an attenuated total reflectance (ATR) device, from 4000 to 500 cm^−1^, at a resolution of 16 cm^−1^, obtaining each spectrum as the average of 12 scans.

### 2.2. Fibre Obtaining and Characterisation

**Figure 2:**
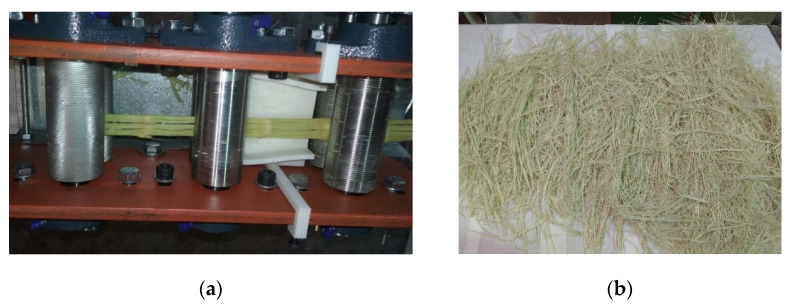
Fibre obtaining: (**a**) Stem passing through rolling mill device and (**b**) fibre bundles.

To obtain the fibres, reeds were cut at the lower end of the stem, separated from the foliage and cut into 50–60 cm fragments for processing. The culms were soaked in water for 1 to 2 days after being crushed to split them lengthwise and remove node protrusions. The fibre bundles were obtained by passing the wet strips through a rolling mill. The lab-made mechanical device (Figure 2), developed for the extraction of giant reed fibres, consist of three pairs of rollers: Knurled pull rollers, smooth pressing rollers and parallel grooved rollers. The rolling process was repeated several times until the fibre bundles were separated, keeping the plant material in water until the process was completed. Afterwards, fibres were air-dried at room conditions.

Obtained fibres were characterised following the above-described procedures to determine their chemical composition and were also subjected to thermogravimetrical analysis (TGA) and infrared spectroscopy (FTIR), performing 9 assays per type of sample for both tests. As reported by several authors, properties and mechanical behaviour of the natural fibres as filler or reinforcement in composite materials depend mainly on their chemical composition. In this regard, a high cellulose content leads to greater stiffness and, consequently, better behaviour as a reinforcement, with higher strength and Young’s modulus [25]. As for hemicellulose, increasing its content increases moisture absorption, accelerates the biodegradation process and decreases tensile strength [25,26], while for lignin, an increase in its content leads to an increase in the moisture gain, failure strain and elongation [25]. Fibres were treated to improve thermal stability and reduce hemicellulose content, by a NaOH treatment (ratio 1 litre of 1 M NaOH per 25 g of fibre) at room temperature for one hour, followed by washing with distillate water [27]; treated fibres were also characterised by the procedures already mentioned.

### 2.3. Composites Obtaining and Characterisation

**Figure 3:**
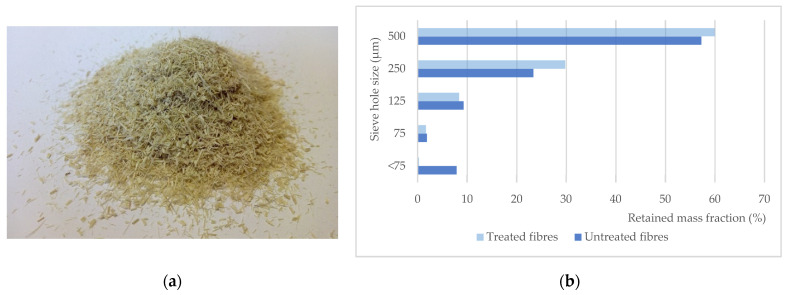
(**a**) Chopped giant reed fibres (2 mm length) and (**b**) particle size distribution.

Fibres were cut into 2 mm length (Figure 3) to produce natural fibre composites (NFC) by dry blending with polymeric matrices and subsequent compression moulding. Two different matrices, polyethylene (Revolve N-461) and polypropylene (PP45), from Matrix Polymers, in powder form, were used to produce composites with 10, 20, 30 and 40% (by weight) fibre content for both treated (ADt) and untreated *Arundo donax* fibres (AD). The chopped fibres were sieved to determine the particle size distribution using 75, 125, 250 and 500 μm sieves. The number of fines, not retained by the 250 μm sieve, is about 19.1% for the untreated fibre and 10.4% for the treated ones. Particles smaller than 125 μm represent a fraction of 9.8% for untreated fibres and only 2% after NaOH treatment; fibre treatment removes fines from the grinding process.

**Figure 4:**
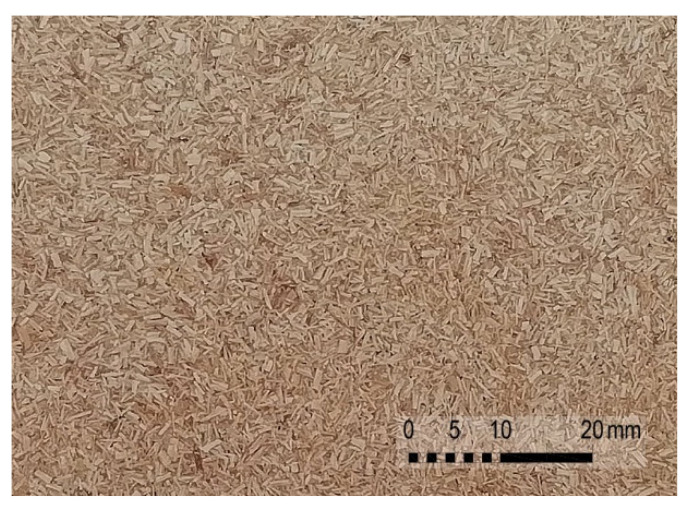
Detail of PE-based composite plate reinforced with 40% fibre.

For each formulation, the dry blend, performed in a laboratory V-mixer, was processed by compression moulding in an aluminium mould to produce square flat plates of 190 × 190 mm and 3 mm thick; the materials were previously dried overnight, polymeric matrices at 40 °C and *Arundo* fibres at 105 °C. Composite plates were moulded in a Collin P200 PM hot press (Collin, Germany), PE at 180 °C and PP at 190 °C and both at 35 bar pressure. Then the moulded parts were machined to obtain test bars with the shape and dimensions defined in UNE-EN ISO 3167, type A for tensile tests, UNE-EN ISO 178 for bending tests and UNE-EN ISO 180 for Izod impact tests. Figure 4 shows the appearance of the composite plates produced.

**Figure 5:**
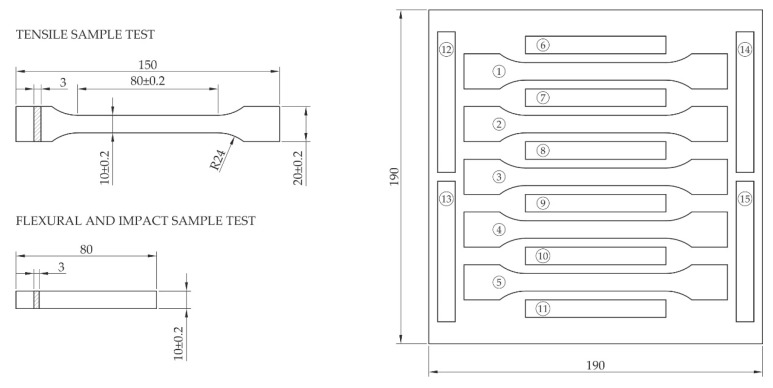
Dimensions and distribution of test samples in the compression-moulded composite plates.

Composites were tested to determine their mechanical behaviour, by flexural, tensile and impact tests. Sample test specimens were distributed on each composite plate as shown in Figure 5. Flexural tests were performed under the recommendations established in UNE-EN ISO 178:2011, while tensile tests follow the standard UNE-EN ISO 527-2:2012, both at a test rate of 10 mm/min. These assays were performed in a universal testing machine from Dongguan Liyi Test Equipment (model LY-1065, China). Impact tests were performed following UNE-EN ISO 180:2001/A2:2013, using unscaled test bars, using a 5.5 J pendulum and impact rate of 3.5 m/s in a Dongguan Liyi Test Equipment (model LY-XJJD 50, China) device. Five samples were tested for each property.

## 3. Results

### 3.1. Plant and Fibre Characterisation

Klason lignin and holocellulose content of different part of giant reed plant and fibres are summarized in Table 1.

Giant reed fibres present a composition similar to that observed in the stems of the plant (24% lignin; 45% cellulose; 35% hemicellulose), which is consistent with the fact that they are obtained from the culms. The NaOH-treated fibres show an increase in cellulose content (from 45% to 57%) and a decrease in hemicellulose content (from 35% to 21%), with no considerable change in lignin content.

From thermogravimetric analysis, it is interesting to note that degradation temperature for fibres is around 230 °C, which is quite close to the degradation temperatures of jute or hemp (205 and 250 °C, respectively) [29]. For instance, the temperatures for a 3% weight loss in *Arundo* fibres are 233.3 ± 5.7 °C and 242.0 ± 5.5 °C for treated ones (not considering moisture); other authors [30] have found this same weight loss at 238 °C for kenaf, 209 °C for sisal or 262 °C for jute, which confirms the potential of *Arundo* fibres for their use in the composites sector. 

**Figure 6:**
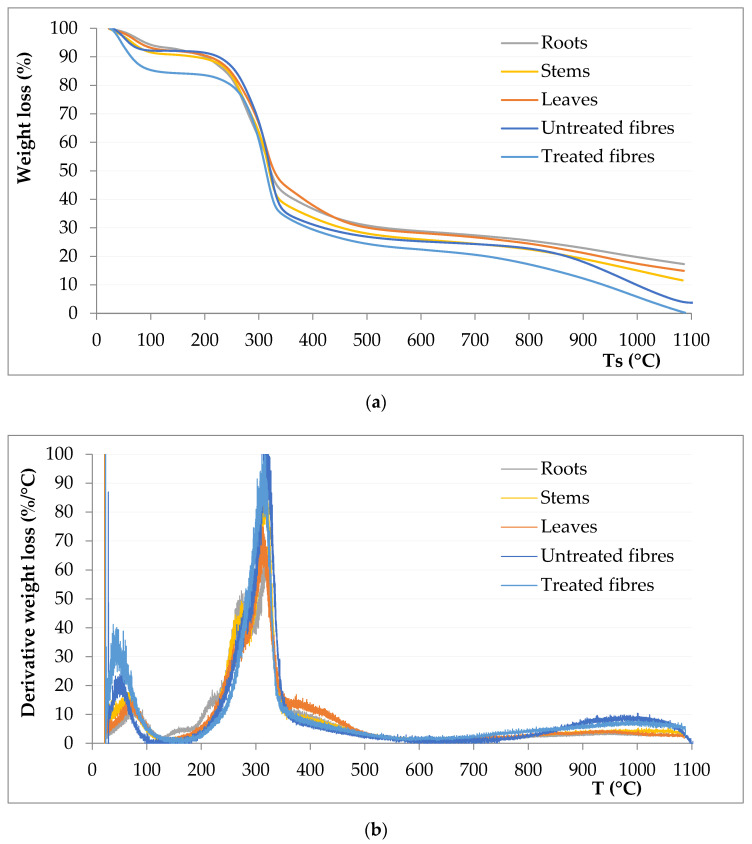
TGA curves for each type of sample: (**a**) Weight loss and (**b**) derivative weight loss.

Thermogravimetric curves (Figure 6) for each part of the plant are very similar to each other; only slight differences can be observed in the moisture content of fibre samples. The main differences are observed in the derivative curves, as for the root and stem samples, two significant and very close peaks are observed; the first one is related to the degradation of hemicellulose, while the second one refers mainly to cellulose. For fibre, it can be observed that degradation starts at slightly higher temperatures than for the rest of the samples.

**Figure 7:**
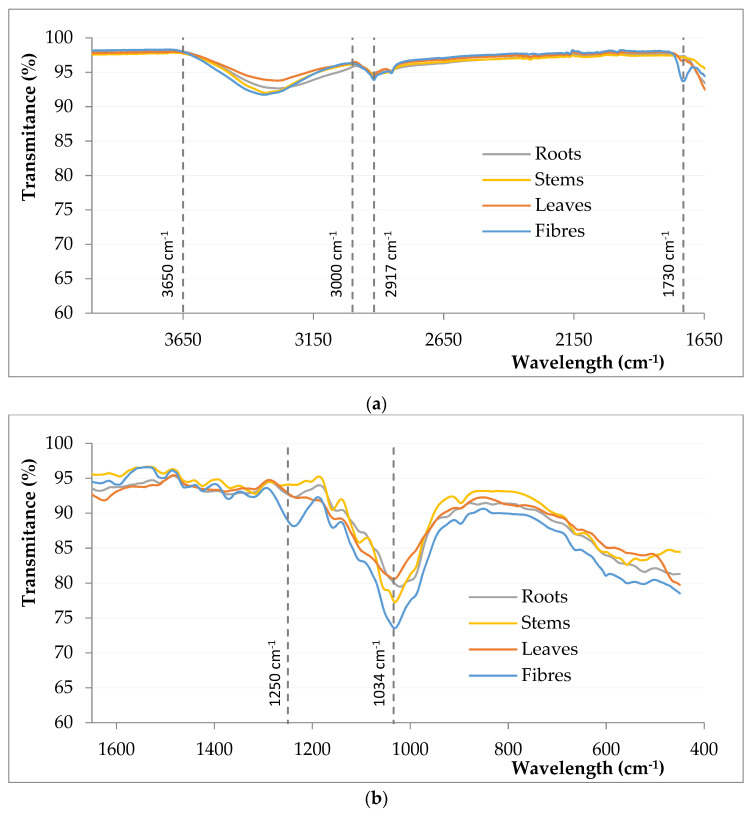
FTIR spectra for different parts of *Arundo donax* plant and untreated fibres (**a**) from 4000 to 1650 cm^−1^ and (**b**) between 1650 and 400 cm^−1^.

The different FTIR spectra obtained for the analysed samples show the characteristic absorption bands of the compounds found in lignocellulosic fibres: Cellulose, hemicellulose and lignin [31]. These compounds are mainly formed by alkenes and aromatic groups, as well as groups containing oxygen atoms, such as esters, ketones or alcohol functional groups. The infrared analysis confirms the chemical composition data obtained by hydrolysis, which show a lower lignin content for the leaves and roots samples, and a higher proportion of cellulose for the fibres. As shown in Figure 7, at 1730 cm^−1^ the peak corresponding to the C=O vibration of ketone or carbonyl groups (mainly associated with the presence of hemicellulose [32], although it may also be due to pectins and waxes) can be observed. This peak occurs for all parts of the plant, although for leaf and root samples, this peak is of very low intensity. At around 1450 cm^−1^ is located the C=C vibration peak of the aromatic structures, which would be given by the lignin. The peak at 1250 cm^−1^ may be due to C–O–C bonds, typical of the polysaccharide structures that compose cellulose. This peak is significantly more pronounced for the fibre samples, which could indicate that the surface structure of the sample has changed.

**Figure 8:**
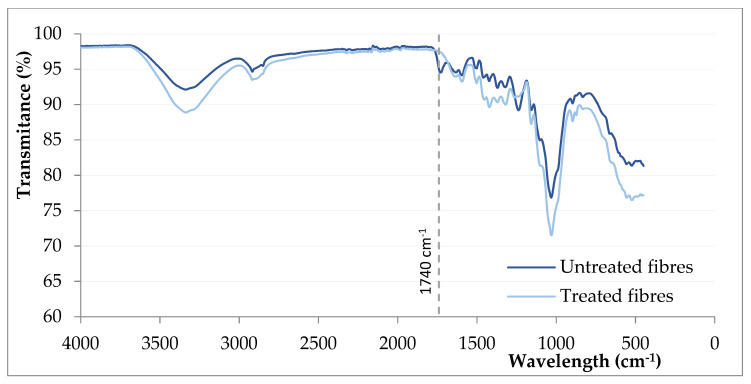
FTIR spectra for untreated and NaOH-treated fibres.

In the comparative study of infrared spectra of untreated and alkaline treated fibres (Figure 8), the only significant difference is observed in the disappearance of the peak at 1740 cm^−1^, associated with the presence of hemicellulose. No appreciable modification of the peak at 1200 cm^−1^ related to lignin is observed, which confirms conclusions from the chemical composition of fibres assays (Table 1).

### 3.2. Composites Characterisation

Table 2 and Table 3 show results obtained from the mechanical tests of *Arundo donax* composites for PE and PP based composites, respectively. The different formulations of NFC were coded according to the following nomenclature: “C” indicates the manufacturing process (by compression moulding), “PE” and “PP” refers to the polymeric matrix, “AD” or “ADt” to the class of fibre, untreated or alkaline treated, and the two numeric digits at the end according to the fibre content by weight. Plates of neat polymeric material were also produced, under the same compression moulding conditions, in order to compare their mechanical performance. The results are given as the mean ± SD of n independent experiments. Statistical comparisons of mechanical performance were conducted using one-way analysis of means (not assuming equal variances), with Tukey’s contrasts for multiple comparisons using R-Commander (R-Studio Software).

**Figure 9:**
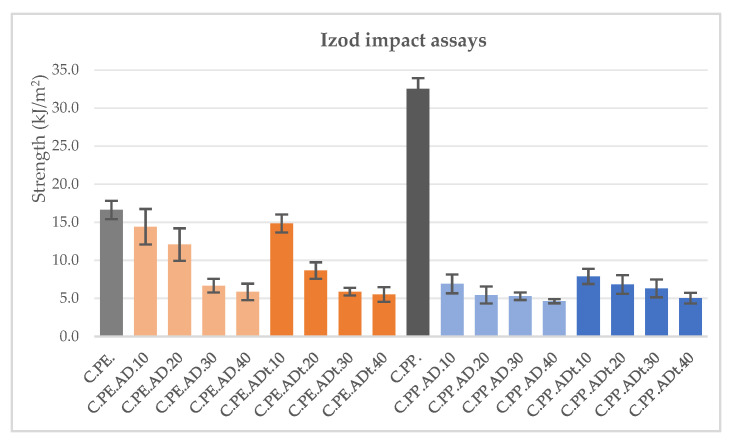
Izod impact assays results for polyethylene and polypropylene based *Arundo donax* composites.

The compression moulding temperature for the matrix-based materials was set at 190 °C due to technical processing limitations, obtaining good mechanical properties, very close to those declared by the manufacturer Matrix Polymers for PP45 neat matrix. When processing the composites with *Arundo* fibre, the flexural and tensile properties are also very acceptable; however, a significant drop in the impact properties is observed, even for the lowest fibre contents. For PE-based composites, the reduction in impact resistance is not as drastic for lower amounts of filler, showing a gradual worsening as fibre content increases (Figure 9).

Density measurement of the composites allows the determination of their specific properties; these become key features to validate the use of NFC in different applications by offering a good balance between strength performance and weight.

**Figure 10:**
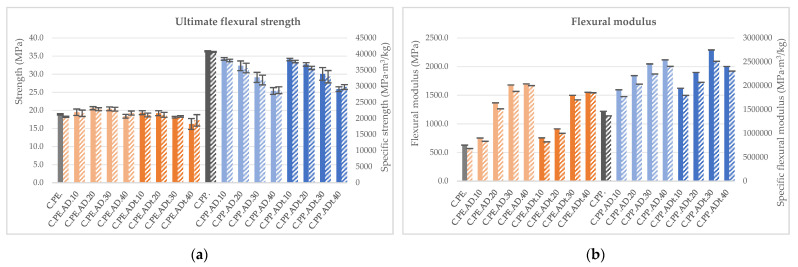
Flexural properties (solid colour) and specific flexural properties (lined) for *Arundo donax* composites: (**a**) Ultimate flexural strength and (**b**) flexural modulus.

As shown in Figure 10a, ultimate flexural strength is not greatly affected by the introduction of fibres into the PE matrix, but it is gradually reduced (up to 30% for composites filled with 40% of fibre) in the case of PP (from 36 MPa for neat PP to 25 MPa for the NFC). In terms of flexural modulus (Figure 10b), it increases considerably, more than twice for PE-based composites (from 628 MPa to near 1700 MPa) and up to 88% for PP-matrix composites (from 1220 to 2290 MPa). The performance is similar for both treated and untreated fibres.

**Figure 11:**
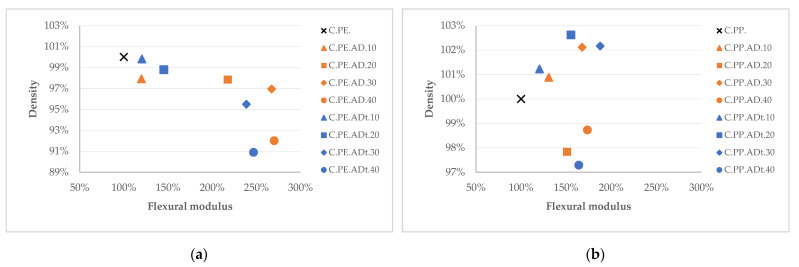
Flexural modulus vs. density variation for (**a**) PE-based and (**b**) PP-based composites.

When comparing the variation of flexural modulus with the variation of density (Figure 11), it is observed that the stiffness increases in all cases for PE-based composites while the materials become lighter as the proportion of fibre increases (up to 9% in weight reduction). The 100% reference values (labelled with a cross) refer to the properties of the neat polymeric matrices and the remaining values refer to the percentage variations of the composite properties with respect to the reference. For the polypropylene NFCs, the stiffness is also improved in all cases, whereas the weight differences are less significant. A similar behaviour is observed for the tensile stiffness tests.

**Figure 12:**
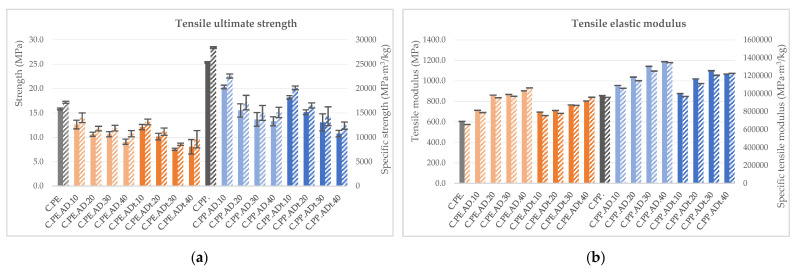
Tensile properties (solid colour) and specific tensile properties (lined) for *Arundo donax* composites: (**a**) Ultimate strength and (**b**) elastic modulus.

As for the flexural loads, the ultimate tensile strength is negatively affected by the introduction of the fibres (Figure 12); a reduction of up to 48% for PE and 42% for PP reinforced with treated fibre is noticed progressively. Regarding the tensile elastic modulus, it rises with increasing giant reed fibre content for both types of polymeric matrices. The highest values are reached for untreated fibre contents of 40% by weight and are about 50% higher than those achieved by the PE neat matrix and by 38% with respect to the PP one (from 605 to 902 MPa and from 857 to 1186 MPa, respectively). 

## 4. Discussion

Results obtained for the chemical characterisation of giant reed fibres are comparable to those of other lignocellulosic fibres used as reinforcement of polymeric matrices and are within the values found in literature for *Arundo donax* fibres [20]. In any case, it is important to highlight that composition data reported for several authors for the giant reed fibres show a wide range of variability (15–33% lignin; 28–45% cellulose; 13–30% hemicellulose) [33,34,35,36,37,38,39]; this evidences that the differences in composition within the same plant species can be significantly affected by the determination methods used and by environmental and process factors affecting the plant growth and the obtained fibre quality such as geographical location of the plant, climate, age, fibre extraction process and experimental conditions. It is interesting to remind at this point that the plants used in this study are not cultivated, but grow without any control, which is one difference with other studies.

Lignin content in this study for different parts of giant reed plants vary from 15% to 21%; the highest concentrations are obtained in roots and stems, while it decreases in leaves, which coincides with the results reported by Pascoal Neto et al. [34]. This can be explained by the fact that lignin is mainly found in the xylem of the plant, deposited in the secondary wall, strengthening the stems and vascular tissues and allowing vertical growth and conduction of water and minerals [40]. In addition, the lignin content of reed is known to increase from the youngest to the oldest parts, thus depending on the maturity stage of the plant [34]. The cellulose content is 21–38%, with the highest concentrations in the stalk, while the hemicellulose content varies from 29% to 34% and is therefore homogeneous in the different parts analysed. The composition of the natural fibres determines the properties of the composite material, with the role of lignin being associated with adhesion mechanisms while the cellulose content has a significant influence on the mechanical performance of the composite [41]. Even if cellulose content is higher in treated fibres than in raw ones, no significant differences are found between composites with treated and untreated fibres. This can be attributed to the fact that not only chemical composition affects the final properties of the composite, but also other properties, such as surface roughness, fibre diameter (length/diameter ratio), moisture absorption, fibre distribution within the matrix, etc., greatly affects the composite performance.

Thermogravimetric analysis tests show a higher thermal stability for the NaOH-treated fibre, with an increase in the temperature of onset of degradation (left limit) of about 20 °C and all other temperatures remaining practically unchanged. In terms of chemical composition, only a decrease in hemicellulose content seems to occur as a consequence of the alkaline treatment, since the weight loss observed for both samples between 220 and 278 °C is lower for the treated fibre. This observation is in agreement with the conclusions drawn from tests to determine the chemical composition of the fibres by quantitative methods. Results from the FTIR spectrum analysis can be also confirmed by the chemical composition data obtained by hydrolysis, which show a lower lignin content for the leaf and root samples, and a higher proportion of cellulose for the fibre.

No references have been found for composites produced with the same combination of thermoplastic matrices and natural fibres used in this work. Although several authors have used thermosetting matrices [19,33,42] or even binder materials of natural origin, such as a resin formulated from castor oil [43,44] or citric acid [45], for the production of giant reed fibre reinforced panels, only Fiore et al. deal with a thermoplastic matrix to produce PLA-based biocomposites reinforced with the *Arundo donax* filler [18].

As observed from the graphs included in the previous section and highlighted in Table 4, impact properties are greatly affected by the introduction of fibres into the composite material, and this may be due to a temperature deficit during the compression moulding process, which makes it difficult for the molten polymer to flow and impregnate the fibres. This worsening of impact resistance is not consistent with the usual improvements found in most references in the literature [46], but is comparable to the results obtained by Lei et al. in the development of recycled HDPE biocomposites filled with different natural fibres [47]. The stiffness values tend to increase as the fibre content rises, while the ultimate strength is significantly reduced for tensile loads and has no considerable change in bending. It should be noted that the NaOH treatment of the fibres does not produce the expected improvement effect on the mechanical properties of the composites, and this may be due to the formation of fibre clusters during the treatment and moulding processes.

According to the statistical inferential study of the composite materials prepared with the PE matrix, the main findings are:

- In terms of impact strength, no differences are observed between composites with the same fibre content, whether treated or untreated.
- Ultimate tensile strength shows a worsening performance trend similar to that observed for the impact strength properties as the fibre content increases, which is in line with the findings of Fiore et al. [18] for PLA composites with *Arundo* fibres. The elastic modulus only shows significant differences with the neat matrix when the proportion of fibre is equal or higher than 20%, obtaining slightly better results with the untreated fibre.
- The incorporation of *Arundo* fibres in the composite material does not seem to affect the ultimate strength and yield strength under bending loads. However, the elastic modulus in bending does show significant differences from 20% untreated fibre content and 30% for the treated ones.
- As shown in Table 5, based on specific properties, the best stiffness results, both in bending and tensile, are obtained for the 40% untreated PE formulation.

From the comparative analysis of the PP matrix-based formulations, the following can be outlined:

- The absorbed impact energy is substantially reduced even for low fibre contents, with no significant differences when fibre content exceeds 20%.
- The tensile behaviour is similar to that observed for composites with PE matrix: The ultimate strength decreases and the elastic modulus gradually increases, which is in accordance with the behaviour observed by Arrakhiz et al. for PP composites reinforced with alpha fibre [48]; no significant differences are observed when the fibre content is increased to more than 30%. The yield strength value remains practically unchanged except for composites reinforced with 40% of NaOH treated fibre.
- Variations in ultimate flexural strength only seem to be relevant for formulations with higher fibre content (30 and 40%). Meanwhile, the flexural stiffness behaviour improves as the fibre content increases, except for 40% treated fibres, where a drop in this property is observed (Table 6).

## 5. Conclusions

Composition of *Arundo donax* L. fibres obtained from residual biomass from invasive specimens is similar to those shown by other cellulosic fibres, as well as their FTIR spectra and thermal behaviour.

Thermal stability increases with NaOH treatment, as shown by the increased degradation temperature of treated fibres, but no significant improvement in mechanical performance is observed.

The introduction of giant reed fibres in PE and PP matrices has been successfully achieved, reaching up to 40% in weight by compression moulding process. These composites show an improvement in flexural and tensile elastic modulus, although with a gradual decrease in the ultimate strength and poor impact properties.

*Arundo donax* L. has shown great potential to be used as reinforcement in polymer composites. The use of these invasive plants provides an added value to the use of natural sourced materials, as a strategy for the control (or eradication in the best of cases) of these species could be outlined, giving a final use to the wastes produced in these campaigns.

From the statistical analysis of the mechanical properties, it can be concluded that no differences are observed between composites with the same fibre content, either for treated or untreated fibres. A decrease in ultimate tensile strength arises with the increase in fibre ratio, while the elastic modulus, both for flexural and tensile tests, is significantly increased for composites with more than 20% of fibre. Trends are similar for both types of matrices, but PE composites with low fibre content show lower reduction for ultimate tensile strength and impact resistance. The future objective of this research work is to combine these type of fibres, coming from the residual biomass generated in invasive species removal campaigns, with thermoplastic polymers, including those obtained from renewable sources, for the preparation of NFC with improved performance through extrusion and injection processes, in addition to studying their biodegradability and recyclability in order to complete the sustainability criteria.

## Figures and Tables

**Table 1:** Chemical composition of *Arundo donax* L.

Section of the Plant	Lignin (%)	Holocellulose (%)	
		Cellulose (%)	Hemicellulose (%)
Roots	19.11 ± 2.08	21.00 ± 1.01	29.57 ± 2.87
Stems	21.11 ± 0.86	37.95 ± 4.59	34.02 ± 1.46
Leaves	15.52 ± 2.00	27.68 ± 3.51	34.09 ± 3.28
Untreated fibres	24.12 ± 1.40	45.16 ± 2.97	35.10 ± 2.80
NaOH-treated fibres	22.21 ± 0.87	56.95 ± 1.68	21.00 ± 1.86

Mean values ± standard deviation of 15 replicates; standard methods for composition determination based in gravimetric methods, such as the ones used here, usually lead to an overestimation of components, thus leading to compositions slightly over 100% [28].

**Table 2:** Mechanical properties of PE-based composites.

Composite	Density	Impact Properties		Tensile Properties (MPa)						Flexural Properties (MPa)					
	(g/cm^3^)	Strength (kJ/m^2^)		Ultimate Strength		E		Yield Strength		Ultimate Strength		E_f_		Yield Strength	
C.PE.	0.921 ^a^	16.6	±1.21 ^a^	15.8	±0.19 ^a^	605.6	±21.88 ^a^	4.6	±0.17 ^a^	18.9	±0.59 ^ab^	628.3	±61.40 ^a^	10.6	±1.52 ^a^	
C.PE.AD.10	0.902 ^a^	14.4	±2.34 ^ab^	12.6	±0.89 ^b^	712.3	±19.00 ^ab^	6.5	±0.38 ^bc^	19.5	±1.17 ^ab^	752.9	±22.30 ^a^	11.9	±0.90 ^a^	
C.PE.AD.20	0.902 ^a^	12.1	±2.13 ^bc^	10.6	±0.42 ^cde^	861.2	±36.4 ^cd^	6.9	±0.20 ^c^	20.6	±1.93 ^b^	1366.8	±210.02 ^b^	11.1	±1.67 ^a^	
C.PE.AD.30	0.893 ^ab^	6.7	±0.91 ^d^	10.6	±0.52 ^d^	867.7	±33.35 ^cd^	7.8	±0.48 ^d^	20.4	±1.04 ^b^	1679.1	±164.32 ^b^	11.0	±1.30 ^a^	
C.PE.AD.40	0.848 ^b^	5.9	±1.08 ^d^	9.1	±0.54 ^cf^	902.7	±99.17 ^d^	7.0	±0.30 ^bcd^	18.4	±1.74 ^ab^	1697.7	±199.43 ^b^	10.5	±1.72 ^a^	
C.PE.ADt.10	0.920 ^a^	14.8	±1.18 ^ab^	12.1	±0.54 ^be^	695.8	±30.77 ^ab^	5.9	±0.48 ^be^	19.4	±0.73 ^ab^	756.5	±69.02 ^a^	12.9	±0.32 ^a^	
C.PE.ADt.20	0.910 ^a^	8.6	±1.09 ^c^	10.2	±0.68 ^cd^	710.7	±45.80 ^b^	6.6	±0.54 ^bc^	19.2	±0.77 ^ab^	912.8	±140.95 ^a^	13.3	±2.22 ^a^	
C.PE.ADt.30	0.880 ^ab^	5.9	±0.50 ^d^	7.6	±0.22 ^g^	764.9	±79.41 ^bc^	5.4	±0.41 ^ae^	18.1	±3.33 ^ab^	1499.0	±298.96 ^b^	11.2	±2.18 ^a^	
C.PE.ADt.40	0.838 ^ab^	5.5	±0.95 ^d^	8.1	±1.49 ^fg^	803.6	±22.76 ^cd^	6.9	±0.89 ^bcd^	16.3	±1.47 ^a^	1551.0	±212.11 ^b^	10.1	±1.08 ^a^	

Values are the mean ± SD of five separate determinations. Means in the same column with the same lowercase letters (^a^, ^b^, ^c^, ^d^…) are not significantly different (*p* > 0.05).

**Table 3:** Mechanical properties of PP-based composites.

Composite	Density	Impact Properties		Tensile Properties (MPa)						Flexural Properties (MPa)					
	(g/cm^3^)	Strength (kJ/m^2^)		Ultimate Strength		E		Yield Strength		Ultimate Strength		E_f_		Yield Strength	
C.PP.	0.893 ^a^	32.5	±1.40 ^a^	25.4	±0.15 ^a^	857.2	±13.80 ^a^	11.1	±0.41 ^a^	36.3	±1.02 ^a^	1220.6	±81.77 ^a^	22.2	±1.76 ^a^	
C.PP.AD.10	0.901 ^a^	6.9	±1.24 ^bc^	20.3	±0.37 ^b^	955.4	±28.22 ^bc^	11.7	±0.66 ^a^	34.2	±0.48 ^ab^	1595.3	±121.32 ^ab^	19.6	±1.65 ^ab^	
C.PP.AD.20	0.906 ^a^	5.4	±1.12 ^bd^	15.5	±1.34 ^c^	1037.8	±29.77 ^cde^	10.4	±0.97 ^a^	32.3	±1.21 ^ac^	1842.2	±246.29 ^bc^	17.8	±1.91 ^b^	
C.PP.AD.30	0.912 ^a^	5.3	±0.49 ^bd^	13.7	±1.38 ^cd^	1142.0	±81.88 ^ef^	10.7	±1.08 ^a^	29.1	±3.05 ^cd^	2046.8	±268,55 ^bc^	18.6	±2.54 ^ab^	
C.PP.AD.40	0.881 ^a^	4.6	±0.27 ^d^	13.3	±0.94 ^de^	1186.7	±29.89 ^f^	10.4	±0.58 ^a^	25.4	±3.94 ^d^	2119.5	±412.60 ^c^	16.2	±3.01 ^b^	
C.PP.ADt.10	0.904 ^a^	7.9	±0.99 ^c^	18.2	±0.34 ^f^	876.2	±43.15 ^ab^	10.7	±0.73 ^a^	34.1	±1.53 ^ab^	1622.8	±111.31 ^b^	17.5	±1.23 ^b^	
C.PP.ADt.20	0.916 ^a^	6.8	±1.22 ^cd^	15.2	±0.48 ^ce^	1019.1	±52.90 ^cd^	10.3	±0.44 ^a^	32.7	±1.00 ^ac^	1897.3	±51.60 ^bc^	18.1	±1.85 ^b^	
C.PP.ADt.30	0.912 ^a^	6.3	±1.17 ^cd^	13.1	±1.75 ^d^	1099.7	±97.62 ^df^	10.0	±0.77 ^ab^	30.1	±2.17 ^bce^	2291.5	±170.05 ^c^	18.2	±2.54 ^b^	
C.PP.ADt.40	0.869 ^a^	5.0	±0.69 ^bd^	10.8	±0.65 ^g^	1066.3	±46.23 ^de^	8.4	±0.39 ^b^	25.9	±4.19 ^de^	2002.3	±348.35 ^bc^	15.8	±2.53 ^b^	

Values are the mean±SD of five separate determinations. Means in the same column with the same lowercase letters (^a^, ^b^, ^c^, ^d^…) are not significantly different (*p* > 0.05).

**Table 4:** Variation of specific properties for all composite formulations with respect to the polymer matrix (improvement highlighted in green and decline in red).

Composite	Density		TENSILE Specific (MPa·m^3^/kg)			FLEXURAL Specific (MPa·m^3^/kg)		
	(g/cm^3^)	Specific Strength (kJ·m/kg)	Ultimate Strength	E	Yield Strength	Ultimate Strength	E_f_	Yield Strength
C.PE.	100.0%	100.0%	100.0%	100.0%	100.0%	100.0%	100.0%	100.0%	
C.PE.AD.10	97.9%	88.6%	81.6%	120.1%	146.7%	105.4%	122.4%	114.7%	
C.PE.AD.20	97.8%	74.3%	68.7%	145.3%	154.8%	111.5%	222.4%	106.6%	
C.PE.AD.30	96.9%	41.4%	69.3%	147.8%	177.4%	111.6%	275.7%	106.6%	
C.PE.AD.40	92.0%	38.3%	62.6%	162.0%	167.7%	105.8%	293.7%	107.0%	
C.PE.ADt.10	99.8%	89.4%	76.6%	115.1%	130.6%	102.8%	120.6%	121.5%	
C.PE.ADt.20	98.8%	52.7%	65.0%	118.8%	147.4%	103.0%	147.1%	126.4%	
C.PE.ADt.30	95.5%	37.0%	50.0%	132.3%	125.2%	100.5%	249.9%	110.4%	
C.PE.ADt.40	90.9%	36.5%	56.0%	146.0%	167.6%	94.7%	271.6%	104.6%	
C.PP.	100.0%	100.0%	100.0%	100.0%	100.0%	100.0%	100.0%	100.0%	
C.PP.AD.10	100.9%	21.0%	79.4%	110.5%	104.7%	93.4%	129.6%	87.2%	
C.PP.AD.20	101.5%	16.5%	60.3%	119.3%	92.7%	87.7%	148.7%	79.0%	
C.PP.AD.30	102.1%	15.9%	52.8%	130.5%	94.4%	78.5%	164.2%	81.9%	
C.PP.AD.40	98.7%	14.4%	53.1%	140.2%	95.1%	70.7%	175.9%	73.9%	
C.PP.ADt.10	101.2%	23.9%	70.9%	101.0%	95.0%	92.7%	131.3%	77.7%	
C.PP.ADt.20	102.6%	20.4%	58.2%	115.9%	90.6%	87.7%	151.5%	79.5%	
C.PP.ADt.30	102.2%	19.0%	50.5%	125.6%	88.5%	81.0%	183.8%	80.2%	
C.PP.ADt.40	97.3%	15.9%	43.7%	127.9%	78.2%	73.2%	168.6%	73.1%	

The 100% reference values refer to the properties of the neat polymeric matrices and the remaining values refer to the percentage variations with respect to the reference.

**Table 5:** Tensile and flexural specific modulus of the PE-based composites.

Composite	E Tensile Specific Modulus (MPa·m^3^/g)		Ef Flexural Specific Modulus (MPa·m^3^/g)	
C.PE.	654.6	±23.71 ^a^	684.6	±68.81 ^a^
C.PE.AD.10	778.9	±21.31 ^bc^	846.1	±39.38 ^a^
C.PE.AD.20	946.1	±44.34 ^de^	1519.6	±216.38 ^b^
C.PE.AD.30	959.8	±39.50 ^de^	1891.8	±207.7 ^bc^
C.PE.AD.40	1032.4	±50.55 ^e^	1991.5	±234.1 ^c^
C.PE.ADt.10	748.1	±33.13 ^ab^	827.5	±893.3 ^a^
C.PE.ADt.20	773.0	±55.61 ^b^	1020.9	±157.8 ^a^
C.PE.ADt.30	866.8	±90.69 ^cd^	1694.1	±297.8 ^bc^
C.PE.ADt.40	943.3	±46.7 ^de^	1791.8	±207.7 ^bc^

Values are the mean±SD of five separate determinations. Means in the same column with the same lowercase letters (^a^, ^b^, ^c^, ^d^…) are not significantly different (*p* > 0.05).

**Table 6:** Tensile and flexural specific modulus of PP-based composites.

Composite	E Tensile Specific Modulus (MPa·m^3^/g)		Ef Flexural Specific Modulus (MPa·m^3^/g)	
C.PP.	959.1	±16.60 ^a^	1371.6	±95.14 ^a^
C.PP.AD.10	1063.5	±31.02 ^ab^	1768.1	±127.32 ^b^
C.PP.AD.20	1149.6	±0.03 ^bcd^	2032.8	±0.25 ^bc^
C.PP.AD.30	1251.5	±0.08 ^de^	2266.9	±0.27 ^cde^
C.PP.AD.40	1287.6	±0.03 ^e^	2496.7	±0.41 ^de^
C.PP.ADt.10	968.7	±0.04 ^a^	1791.0	±0.11 ^b^
C.PP.ADt.20	1111.4	±0.05 ^bc^	2085.0	±0.05 ^bd^
C.PP.ADt.30	1194.8	±0.09 ^ce^	2506.6	±0.17 ^e^
C.PP.ADt.40	1172.9	±0.05 ^be^	2318.7	±0.35 ^cde^

Values are the mean ± SD of five separate determinations. Means in the same column with the same lowercase letters are not significantly different (*p* > 0.05).

## Data Availability

Not applicable.

## References

[1] Lowe S., Brown M., Boudejelas D.P. (2020). 100 of the World’s Worst Invasive Alien Species: A Selection From The Global Invasive Species Database. Encyclopedia of Biological Invasions.

[2] Global Invasive Species Database Species profile: Arundo donax. http://www.iucngisd.org/gisd/species.php?sc=112.

[3] Sanz Elorza M., Dana Sáncehz E.D., Sobrino Vesperinas E. (2004). Atlas de las Plantas Alóctonas Invasoras en España.

[4] Silva L., Ojeda Land E., Rodríguez-Luengo J.L. (2008). Flora y Fauna Terrestre Invasora en la Macaronesia—Top. 100 en Azores, Madeira y Canarias.

[5] Jiménez-Ruiz J., Hardion L., Del Monte J.P., Vila B., Santín-Montanyá M.I. (2021). Monographs on invasive plants in Europe N° 4: *Arundo donax* L.. Bot. Lett..

[6] Giessow J., Casanova J., Leclerc R., MacArthur R., Fleming G. (2011). Arundo Donax. Distribution and Impact Report.

[7] Coffman G.C., Ambrose R.F., Rundel P.W. (2010). Wildfire promotes dominance of invasive giant reed (*Arundo donax*) in riparian ecosystems. Biol. Invasions.

[8] Antonetti C., Bonari E., Licursi D., Di Nasso N.N., Galletti A.M.R. (2015). Hydrothermal conversion of giant reed to furfural and levulinic acid: Optimization of the process under microwave irradiation and investigation of distinctive agronomic parameters. Molecules.

[9] Proietti S., Moscatello S., Fagnano M., Fiorentino N., Impagliazzo A., Battistelli A. (2017). Chemical composition and yield of rhizome biomass of *Arundo donax* L. grown for biorefinery in the Mediterranean environment. Biomass Bioenergy.

[10] Mirza N., Mahmood Q., Pervez A., Ahmad R., Farooq R., Shah M.M., Azim M.R. (2010). Phytoremediation potential of *Arundo donax* in arsenic-contaminated synthetic wastewater. Bioresour. Technol..

[11] Alwared A.I., Jaeel A.J., Ismail Z.Z. (2020). New application of eco-friendly biosorbent giant reed for removal of reactive dyes from water followed by sustainable path for recycling the dyes-loaded sludge in concrete mixes. J. Mater. Cycles Waste Manag..

[12] Calabrese L., Piperopoulos E., Fiore V. (2020). Arundo donax fibers as green materials for oil spill recovery. Biofibers and Biopolymers for Biocomposites: Synthesis, Characterization and Properties.

[13] Martínez Gabarrón A., Flores Yepes J.A., Pastor Pérez J.J., Berná Serna J.M., Arnold L.C., Sánchez Medrano F.J. (2014). Increase of the flexural strength of construction elements made with plaster (calcium sulfate dihydrate) and common reed (*Arundo donax* L.). Constr. Build. Mater..

[14] Ismail Z.Z., Jaeel A.J. (2014). A novel use of undesirable wild giant reed biomass to replace aggregate in concrete. Constr. Build. Mater..

[15] Guillamet G.V. (2019). Arundo donax structures as economic and ecological formwork for concrete shells. Proceedings of the International fib Symposium on Conceptual Design of Structures.

[16] Andreu-Rodriguez J., Medina E., Ferrandez-Garcia M.T., Ferrandez-Villena M., Ferrandez-Garcia C.E., Paredes C., Bustamante M.A., Moreno-Caselles J. (2013). Agricultural and Industrial Valorization of *Arundo donax* L.. Commun. Soil Sci. Plant. Anal..

[17] Ferrandez-García M.T., Ferrandez-Garcia A., Garcia-Ortuño T., Ferrandez-Garcia C.E., Ferrandez-Villena M. (2020). Assessment of the physical, mechanical and acoustic properties of *Arundo donax* L. biomass in low pressure and temperature particleboards. Polymers.

[18] Fiore V., Botta L., Scaffaro R., Valenza A., Pirrotta A. (2014). PLA based biocomposites reinforced with *Arundo donax* fillers. Compos. Sci. Technol..

[19] Scalici T., Fiore V., Valenza A. (2016). Effect of plasma treatment on the properties of Arundo Donax L. leaf fibres and its bio-based epoxy composites: A preliminary study. Compos. Part. B Eng..

[20] Fiore V., Scalici T., Valenza A. (2014). Characterization of a new natural fiber from *Arundo donax* L. as potential reinforcement of polymer composites. Carbohydr. Polym..

[21] García-Ortuño T., Andréu-Rodríguez J., Ferrández-García M.T., Ferrández-Villena M., Ferrández-García C.E. (2011). Evaluation of the physical and mechanical properties of particleboard made from giant reed (*Arundo donax* L.). BioResources.

[22] Fiore V., Scalici T., Vitale G., Valenza A. (2014). Static and dynamic mechanical properties of Arundo Donax fillers-epoxy composites. Mater. Des..

[23] (1977). Standard Test. Methods for Lignin in Wood—D 1106-56. 1977a.

[24] Browing B.L. (1967). Methods of wood chemistry. Polym. Phys..

[25] Komuraiah A., Kumar N.S., Prasad B.D. (2014). Chemical Composition of Natural Fibers and its Influence on their Mechanical Properties. Mech. Compos. Mater..

[26] Velásquez Restrepo S.M., Peaéz Arroyave G.J., Giraldo Vásquez D. (2016). Uso de fibras vegetales en materiales compuestos de matriz polimérica: Una revisión con miras a su aplicación en el diseño de nuevos productos. SENA Cent. Nac. Asist. Técnica Ind..

[27] Benítez A.N., Monzón M.D., Angulo I., Ortega Z., Hernández P.M., Marrero M.D. (2013). Treatment of banana fiber for use in the reinforcement of polymeric matrices. Meas. J. Int. Meas. Confed..

[28] Mccleary B.V., Cox J. (2017). Evolution of a Definition for Dietary Fiber and Methodology to Service this Definition. Luminacoids Res..

[29] De Rosa I.M., Kenny J.M., Puglia D., Santulli C., Sarasini F. (2010). Morphological, thermal and mechanical characterization of okra (Abelmoschus esculentus) fibres as potential reinforcement in polymer composites. Compos. Sci. Technol..

[30] Poletto M., Ornaghi Jnior H.L., Zattera A.J. (2015). Thermal Decomposition of Natural Fibers: Kinetics and Degradation Mechanisms. Reactions and Mechanisms in Thermal Analysis of Advanced Materials.

[31] Satyanarayana K.G., Guimarães J.L., Wypych F. (2007). Studies on lignocellulosic fibers of Brazil. Part I: Source, production, morphology, properties and applications. Compos. Part. A Appl. Sci. Manuf..

[32] Oliveira M.A.S., Pickering K.L., Sunny T., Lin R.J.T. (2021). Treatment of hemp fibres for use in rotational moulding. J. Polym. Res..

[33] Fuentes R., Gordillo M., Mendoza M., Amezquita F. (2011). Mechanical response of composites with arundo donax as enforcer to polyster resins. Nat. Tecnol..

[34] Neto C.P., Seca A., Nunes A.M., Coimbra M.A., Domingues F., Evtuguin D., Silvestre A., Cavaleiro J.A.S. (1997). Variations in chemical composition and structure of macromolecular components in different morphological regions and maturity stages of *Arundo donax*. Ind. Crops Prod..

[35] Krička T., Matin A., Bilandžija N., Jurišić V., Antonović A., Voća N., Grubor M. (2017). Biomass valorisation of *Arundo donax* L., Miscanthus × giganteus and Sida hermaphrodita for biofuel production. Int. Agrophys..

[36] Temiz A., Akbas S., Panov D., Terziev N., Alma M.H., Parlak S., Kose G. (2013). Chemical composition and efficiency of bio-oil obtained from giant cane (arundo donax l.) as a wood preservative. BioResources.

[37] Ahmed M.J. (2016). Potential of *Arundo donax* L. stems as renewable precursors for activated carbons and utilization for wastewater treatments: Review. J. Taiwan Inst. Chem. Eng..

[38] De Bari I., Liuzzi F., Villone A., Braccio G. (2013). Hydrolysis of concentrated suspensions of steam pretreated *Arundo donax*. Appl. Energy.

[39] Komolwanich T., Tatijarern P., Prasertwasu S., Khumsupan D., Chaisuwan T., Luengnaruemitchai A., Wongkasemjit S. (2014). Comparative potentiality of Kans grass (Saccharum spontaneum) and Giant reed (*Arundo donax*) as lignocellulosic feedstocks for the release of monomeric sugars by microwave/chemical pretreatment. Cellulose.

[40] Ávalos García A., Pérez-Urria Carril E. (2009). Metabolismo secundario de plantas. REDUCA.

[41] Shalwan A., Yousif B.F. (2013). In state of art: Mechanical and tribological behaviour of polymeric composites based on natural fibres. Mater. Des..

[42] Prathap K.A. (2019). Comparative Study on the Mechanical Properties of Arundo Donax Epoxy Composites with Bamboo Epoxy Composites. Int. J. Eng. Res..

[43] Baquero Basto D., Alarcón J.M., Sánchez Cruz M. (2018). Experimental characterization of composite panels made with Arundo Dónax fibers and vegetable resin. Sci. Tech. Año XXII.

[44] Monsalve Alarcón J., Sánchez Cruz M., Baquero Bastos D. (2018). Evaluation of the physical and mechanical properties of caña brava (*Arundo donax*) reinforced panels. INGE CUC.

[45] Ferrandez-Garcia M.T., Ferrandez-Garcia C.E., Garcia-Ortuño T., Ferrandez-Garcia A., Ferrandez-Villena M. (2019). Experimental evaluation of a new giant reed (*Arundo donax* L.) composite using citric acid as a natural binder. Agronomy.

[46] Sanjay M.R., Madhu P., Jawaid M., Senthamaraikannan P., Senthil S., Pradeep S. (2018). Characterization and properties of natural fiber polymer composites: A comprehensive review. J. Clean. Prod..

[47] Lei Y., Wu Q., Yao F., Xu Y. (2007). Preparation and properties of recycled HDPE/natural fiber composites. Compos. Part. A Appl. Sci. Manuf..

[48] Arrakhiz F.Z., Elachaby M., Bouhfid R., Vaudreuil S., Essassi M., Qaiss A. (2012). Mechanical and thermal properties of polypropylene reinforced with Alfa fiber under different chemical treatment. Mater. Des..

